# Utility of Flexion and Extension MRI for Evaluating Isolated Cervical Spinal Cord Lesions: A Case Series

**DOI:** 10.7759/cureus.46932

**Published:** 2023-10-12

**Authors:** Zeinab Awada, Sami Saba, Asaff Harel

**Affiliations:** 1 Neurology, Staten Island University Hospital and Lenox Hill Hospital, New York, USA; 2 Neurology, Lenox Hill Hospital/Donald and Barbara Zucker School of Medicine, New York, USA

**Keywords:** degenerative myelopathy, degenerative spine disease, intrinsic cord lesion, intramedullary lesion, flexion and extension mri, compressive myelopathy

## Abstract

The diagnosis of isolated spinal cord lesions is often challenging in clinical practice, and it is not uncommon for the etiology of such isolated lesions to remain unclear despite extensive workup and investigations. Magnetic resonance imaging (MRI) is extensively utilized for assessing spinal cord disease, despite certain radiological patterns suggesting certain pathologies, diagnostic uncertainty remains. Development of adjunct tests and techniques, radiographic or otherwise, is needed. Here, we present two cases in which flexion-extension cervical spine MRIs improved diagnostic ability by demonstrating dynamic cervical cord compression as an etiology for isolated intramedullary cervical spinal cord lesions.

## Introduction

Magnetic resonance imaging (MRI) is the preferred imaging modality for the spinal cord, but isolated spinal cord lesions presenting as hyperintense intramedullary signals on T2-weighted imaging generally do not confer enough specificity to make a diagnosis. Clinical evaluation is paramount and remains the cornerstone of the workup of spinal cord lesions, as it helps in narrowing the differential diagnosis. Spinal cord lesions can occur in the setting of neuroinflammatory diseases such as idiopathic transverse myelitis and multiple sclerosis, metabolic conditions, neoplastic disease, or intrinsic structural pathologies. However, extrinsic compression is a common cause of intramedullary T2 signal abnormality and may not always be readily apparent on routine spine imaging. Flexion-extension studies may demonstrate intermittent spinal cord compression, and this should be considered in the differential of isolated cervical cord lesions.

## Case presentation

Case one

**Figure 1:**
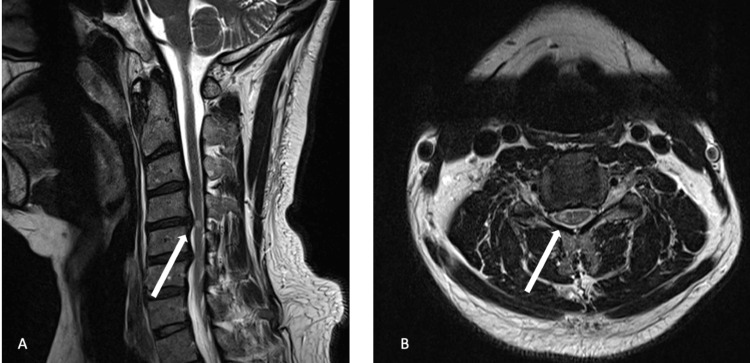
Sagittal (A) and axial (B) T2-weighted MRI of the cervical spine with neutral neck position showing abnormal T2 prolongation involving the right spinal cord at the level of the C4-C5 disc.

A 40-year-old man with no significant past medical history was referred to the neurology clinic for a three-year history of constant, non-painful numbness affecting both feet, up to the ankles bilaterally. His symptoms were worsening gradually over the previous year. The neurological examination was significant for mildly reduced sensation for light touch and temperature, with moderately reduced vibration and proprioception in a stocking-like distribution up to the lower shins, and with mild lower extremity hyperreflexia. Cranial nerves, motor power, and gait were all normal. MRI of the brain and thoracic spine were unremarkable. MRI of the cervical spine showed an isolated T2-hyperintense lesion involving the right spinal cord at the level of the C4-C5 disc associated with mild enhancement that was deemed potentially suggestive of neuroinflammatory etiology (Figure 1). There was mild spinal stenosis at that level with bilateral hypertrophic facets, but no evidence of cord compression.

Extensive workup including infectious, nutritional, and autoimmune panel was negative (hemoglobin A1c, thyroid-stimulating hormone, vitamin B1, vitamin B6, vitamin B12, folic acid, methylmalonic acid, erythrocyte sedimentation rate, antinuclear antibody, antineutrophil cytoplasmic antibodies, Sjögren’s syndrome antibodies, angiotensin-converting enzyme, heavy metal screen, anti-myelin-associated glycoprotein, anti-ganglioside, Lyme serology, copper, cryoglobulin, and homocysteine). The Mayo serum autoimmune myelopathy panel (Mayo Labs Test ID: MAS1) was negative. Cerebrospinal fluid (CSF) analysis demonstrated a mildly elevated protein of 54 mg/dL (reference range = 15-45 mg/dL), with no pleocytosis and absent oligoclonal bands. Nerve conduction studies and electromyography showed reduced right radial sensory amplitude, absent sural, and superficial fibular responses suggestive of peripheral neuropathy. Further workup of neuropathy demonstrated positive antibodies to trisulfated heparin disaccharide suggestive of peripheral neuropathy.

**Figure 2:**
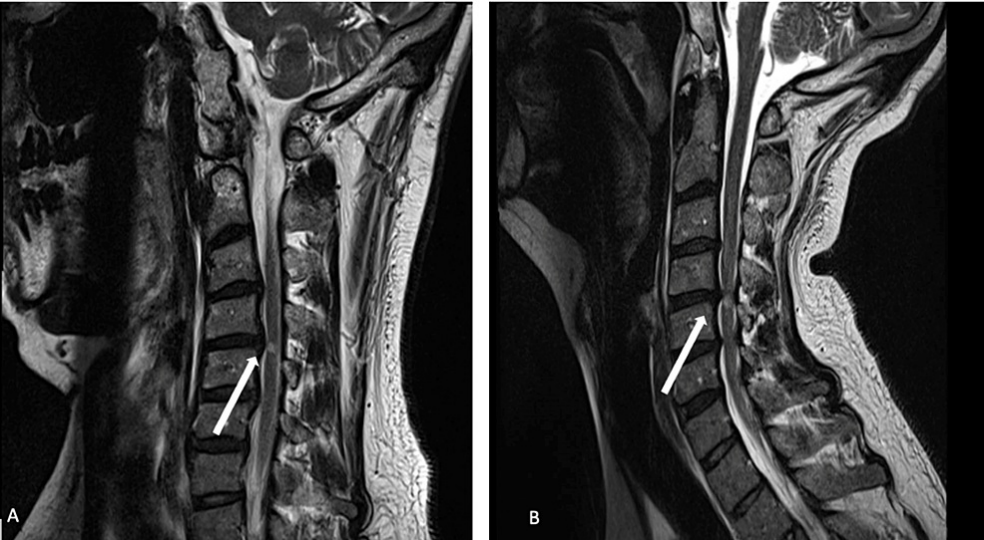
Sagittal T2-weighted MRI of the cervical spine during neck flexion (A) and neck extension (B) showing significant narrowing of the canal and effacement of the cord at the level of the signal abnormality at C4-C5 during neck extension.

While peripheral neuropathy was deemed likely to be the cause of the patient’s symptoms, the etiology of the patient’s isolated spinal cord lesion remained unclear. Given the presence of mild spinal stenosis without cord compression on MRI with a neutral neck position, a dynamic MRI of the cervical spine with neck flexion and extension was performed (Figure 2). The extension study demonstrated worsened central canal stenosis and effacement of the cord at the level of the signal abnormality at C4-C5, while the flexion study was similar to the neutral MRI. This confirmed dynamic compression of the cervical spinal cord causing intramedullary hyperintensity and lower extremity hyperreflexia. The patient was referred to neurosurgery, with a decision to defer surgical intervention unless clinical deterioration was observed.

Case two

A 46-year-old woman with a history of rheumatoid arthritis presented to the neurology clinic complaining of neck pain radiating to the right upper extremity associated with an unsteady gait. The patient reported that the pain originally began 10 years prior, following a motor vehicle accident, and initially subsided with physical therapy. The pain then became worse and more persistent over the previous two years radiating to the right upper extremity and associated with weakness in the right arm and hand. The neurologic examination was significant for symmetric hyperreflexia in the lower extremities and extensor plantar response on the right, in addition to mild weakness with right wrist extension and finger abduction.

**Figure 3:**
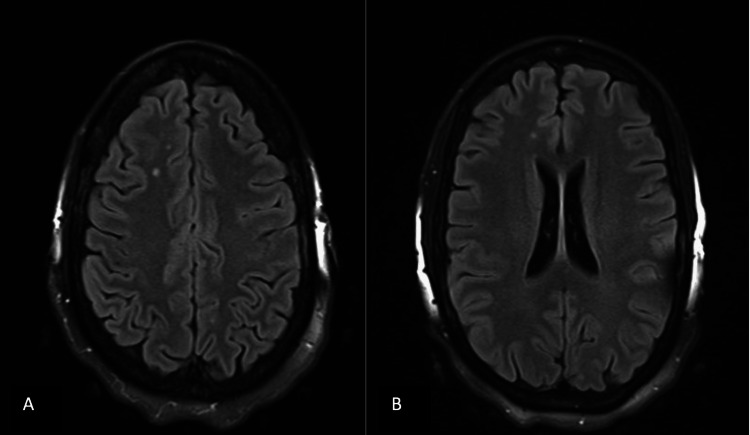
Axial T2 fluid-attenuated inversion recovery brain MRI with mild scattered nonspecific small T2 hyperintensities (A and B).

**Figure 4:**
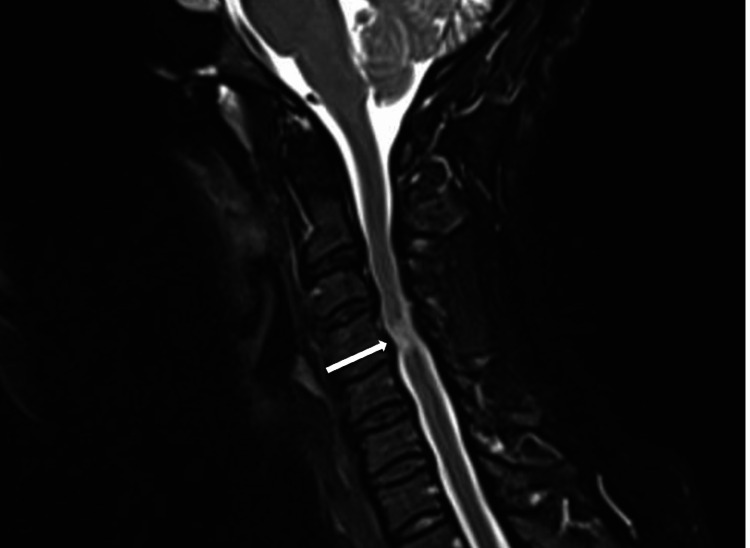
Sagittal T2-weighted MRI of the cervical spine with neutral neck position showing cord signal abnormality at C4-C5 with cord atrophy at that level without obvious extrinsic cord compression.

**Figure 5:**
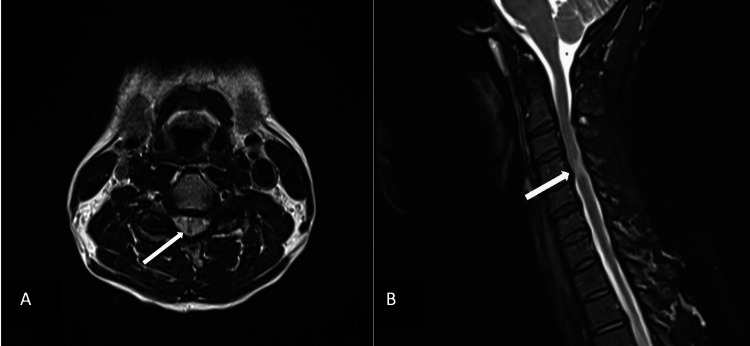
Axial (A) T2-weighted MRI of the cervical spine showing symmetric bilateral lateral cord signal abnormality at C4-C5. Sagittal (B) T2-weighted MRI of the cervical spine with neutral neck position showing cord signal abnormality at C4-C5 with cord atrophy at that level without obvious extrinsic cord compression.

**Figure 6:**
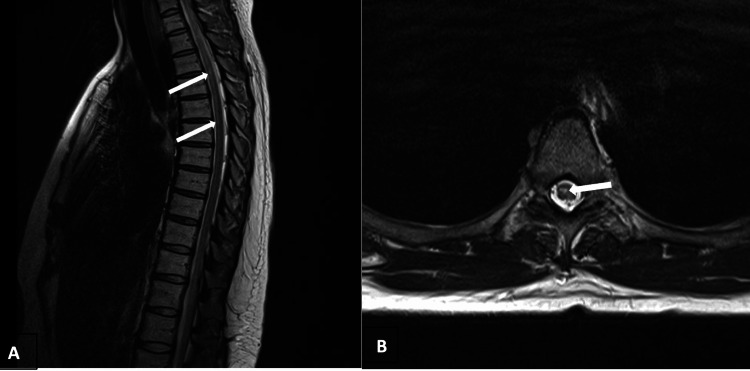
Sagittal (A) and axial T2-weighted MRI of the thoracic spine showing linear T2 signal hyperintensity in the right paracentral spinal cord extending from T2 through T5.

Brain MRI demonstrated scattered nonspecific small T2 hyperintensities (Figure 3). MRI of the cervical spine showed symmetric bilateral lateral cord lesions at C4-C5 and cord atrophy at that level, with congenital stenosis but without cord compression (Figures 4, 5). These changes were felt by the radiologist to be inflammatory in nature. MRI of the thoracic spine revealed linear T2 signal hyperintensity in the right paracentral spinal cord extending from T2 through T5 (Figure 6) of unclear etiology.

Workup including aquaporin-4 antibody, double-stranded DNA, Sjogren’s syndrome antibodies (SSA and SSB), antinuclear antibody (ANA), and Mayo serum autoimmune myelopathy panel (Mayo Labs Test ID: MAS1) was unrevealing. Vitamin B12, vitamin D, ceruloplasmin, and complement levels were within normal limits. Serum copper levels were minimally elevated which normalized on repeat testing. CSF analysis was unremarkable, demonstrating the absence of oligoclonal bands. Given the unrevealing workup, the patient was observed without intervention.

**Figure 7:**
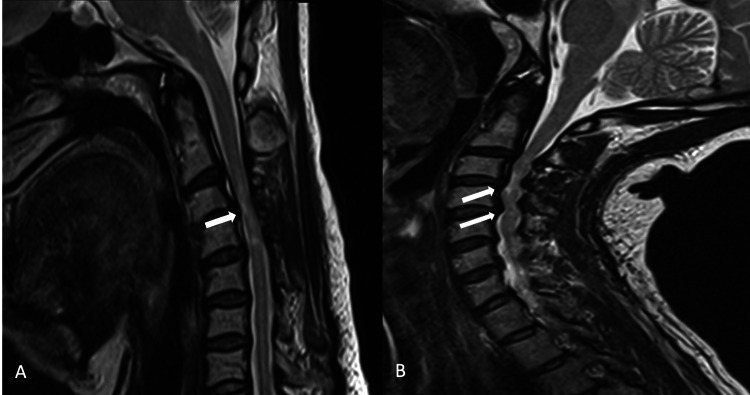
Sagittal T2-weighted MRI of the cervical spine during neck flexion (A) and extension (B) showing spinal cord compression at C3-C4 and C4-C5 upon extension.

Several months later, she developed left hand, torso, and left leg numbness. Given the progression of her symptoms and concern for congenital stenosis, a repeat MRI of the cervical spine was performed, this time with neck flexion and extension protocol, which demonstrated stability of the T2 hyperintensities, but with substantial spinal cord compression at C3-C4 and C4-C5 upon neck extension (Figure 7). As the cervical spinal cord lesions were now deemed likely due to compression, the patient was referred to a spine surgeon and underwent C3-C5 anterior cervical discectomy and fusion. This led to substantial improvement in her symptoms, and she has been clinically and radiographically stable in the three years since the procedure.

## Discussion

Degenerative cervical myelopathy, an age-related condition of the cervical spine, can cause significant neurologic dysfunction. MRI plays an essential role in the evaluation of suspected myelopathy and helps with identifying underlying etiology. Hyperintense intramedullary signals on T2-weighted imaging are nonspecific and may be due to ischemic, infectious, inflammatory, demyelinating causes [1], as well as extrinsic compression. Thus, this can present a diagnostic challenge, as seen in the above-described cases.

In the first case, the patient’s clinical presentation was typical for neuropathy, and was highly atypical for a demyelinating process such as multiple sclerosis (MS), with absent lesions on brain MRI. The cord lesion noted did not fully explain the patient’s symptoms. However, given the preserved and relatively increased reflexes noted on the examination, a superimposed myelopathy was suspected. The patient’s initial MRI did not clearly demonstrate compression; however, dynamic MRI with flexion and extension demonstrated compression upon extension which explained the hyperintense signal within the cord. Eventually, further evaluation led to an underlying etiology for the neuropathy which was the most likely cause of his symptoms. Investigation for inflammatory etiologies of his spinal cord lesion, which was ultimately deemed a “red herring,” led to delayed testing and diagnosis of his neuropathy.

In the second case, although the hyperintense lesions in the spinal cord led to concern of demyelinating disease, brain MRI and CSF analysis were not suggestive of MS or any other central nervous system neuroimmune condition. Dynamic cervical MRI with flexion and extension demonstrated cervical cord compression that was not apparent on the initial MRI, and the patient had significant improvement in symptoms after surgical decompression.

Compressive myelopathy typically exhibits focal T2 hyperintensity in the cord at the level of spinal stenosis with or without associated enhancement [2]. As such, it is important to rule out compressive etiology when investigating an intrinsic isolated spinal cord lesion before an invasive workup. The use of dynamic MRI in cervical spine myelopathy has been previously studied but is not performed routinely due to the additional time, cost, and resources required [3]. Despite this, the potential importance of flexion-extension MRI in the explication of cervical cord compression has long been recognized. Studies have demonstrated that dynamic MRI reveals a greater incidence of spinal cord compression compared to static examination, and extension MRI was useful for the diagnosis of posterior compression [4]. Additionally, neck extension can significantly increase the severity of cord compression compared to the standard supine position [5], as observed in our cases. Moreover, it has also been shown that extension MRI can help to identify significant cervical canal stenosis that is partially or completely absent on neutral, and flexion MRI permits better visualization of high-intensity intrinsic cord lesions on T2-weighted sequences [6]. Therefore, it is reasonable to consider dynamic spinal cord imaging when investigating spinal cord hyperintensities in the appropriate clinical setting, as it will provide a valuable diagnostic tool in the evaluation of spinal cord lesions.

## Conclusions

This case series highlights the need for considering compressive etiology for isolated intramedullary cervical spinal cord hyperintense lesions before more invasive diagnostic testing. Flexion and extension MRI of the cervical spine can be used to evaluate isolated cervical spinal cord lesions seen on static MRI before further workup for other causes such as demyelinating disease. While extension exacerbates spinal compression due to the reduction in spinal canal dimensions, flexion can also intensify certain compression scenarios mainly in severe degenerative disc disease. Integrating flexion and extension MRI can accelerate the detection of compressive causes, minimizing invasive procedures and enhancing patient outcomes via timely intervention.

## References

[1] Guppy KH, Hawk M, Chakrabarti I, Banerjee A (2009). The use of flexion-extension magnetic resonance imaging for evaluating signal intensity changes of the cervical spinal cord. J Neurosurg Spine.

[2] Mohajeri Moghaddam S, Bhatt AA (2018). Location, length, and enhancement: systematic approach to differentiating intramedullary spinal cord lesions. Insights Imaging.

[3] Joaquim AF, Baum GR, Tan LA, Riew KD (2019). Dynamic cord compression causing cervical myelopathy. Neurospine.

[4] Stamm S, McClellan JW 3rd, Knierim A, Suiter IP, Riew KD (2013). Dynamic MRI reveals soft-tissue compression causing progressive myelopathy in postlaminectomy patients: a report of three cases. JBJS Case Connect.

[5] Bartlett RJ, Hill CA, Rigby AS, Chandrasekaran S, Narayanamurthy H (2012). MRI of the cervical spine with neck extension: is it useful?. Br J Radiol.

[6] Zeitoun D, El Hajj F, Sariali E, Catonné Y, Pascal-Moussellard H (2015). Evaluation of spinal cord compression and hyperintense intramedullary lesions on T2-weighted sequences in patients with cervical spondylotic myelopathy using flexion-extension MRI protocol. Spine J.

